# Identification of *TRAPPC9* and *BAIAP2* Gene Polymorphisms and Their Association With Fat Deposition-Related Traits in Hu Sheep

**DOI:** 10.3389/fvets.2022.928375

**Published:** 2022-07-05

**Authors:** Panpan Cui, Weimin Wang, Deyin Zhang, Chong Li, Yongliang Huang, Zongwu Ma, Xiaojuan Wang, Liming Zhao, Yukun Zhang, Xiaobin Yang, Dan Xu, Jiangbo Cheng, Xiaolong Li, Xiwen Zeng, Yuan Zhao, Wenxin Li, Jianghui Wang, Changchun Lin, Bubo Zhou, Jia Liu, Rui Zhai, Xiaoxue Zhang

**Affiliations:** ^1^College of Animal Science and Technology, Gansu Agricultural University, Lanzhou, China; ^2^The State Key Laboratory of Grassland Agro-Ecosystems, College of Pastoral Agriculture Science and Technology, Lanzhou University, Lanzhou, China

**Keywords:** Hu sheep, *TRAPPC9*, *BAIAP2*, fat deposition related traits, qRT-PCR

## Abstract

Fat deposition is an important economic trait that is closely related to feed efficiency and carcass performance in livestock. In this study, the fat deposition-related traits of 1,293 Hu sheep were measured and descriptive statistical analysis was conducted. The results showed that the coefficient of variation of all fat deposition-related traits was higher than 24%. In addition, single nucleotide polymorphisms and the expression characteristics of *TRAPPC9* (encoding trafficking protein particle complex subunit 9) and *BAIAP2* (encoding brain-specific Angiogenesis inhibitor 1-associated protein 2) genes in Hu sheep were detected using PCR amplification, Sanger sequencing, KASPar genotyping, and quantitative real-time reverse transcription PCR (qRT-PCR). The associations between SNPs and fat deposition-related traits were also analyzed. Two intronic mutations, *TRAPPC9* g.57654 A > G and *BAIAP2* g.46061 C > T, were identified in Hu sheep. The result of association analysis showed that *TRAPPC9* g.57654 A > G and *BAIAP2* g.46061 C > T were both significantly associated with the weight of tail fat, tail fat relative weight (body weight), and tail fat relative weight (carcass) (*P* < 0.05). Comprehensive effects analysis showed that there were significant differences between the combined genotypes and tail fat and perirenal fat deposition. Moreover, qRT-PCR analysis showed that *TRAPPC9* and *BAIAP2* are widely expressed, and their expression levels were significantly higher in the small-tail group compared with those in the big-tail group (*P* < 0.01). These results provided important candidate molecular markers that could be used in strategies to reduce tail fat deposition in Hu sheep.

## Introduction

Hu sheep are one of the important livestock breeds in Taihu Lake Basin region of China. They have the advantages of high reproductive performance (long estrus period, average litter size 2.06), fast growth and development performance, strong environmental adaptability, and good lactation performance (1, 2). Nowadays, sheep can be classified into five types on the basis of their tail size: short fat-tailed sheep, long fat-tailed sheep, short thin-tailed sheep, long thin-tailed sheep, and fat-rumped sheep (3). The important fat-tailed breed of sheep was first recorded 5,000 years ago (4). Hu sheep belong to short fat-tailed sheep (3). Adipose tissue plays a vital role in maintaining the balance of homeostatic metabolic processes in domestic animals. And it is found in various parts of the sheep body, including the perirenal, mesentery, and tail. Tail fat is the most typical type of deposited fat (5). During severe conditions, such as food scarcity resulting from migration, drought, and winter, tail fat can provide energy (6). Fat has added value to humans as it can provide high-energy food during droughts and famines (4). Currently, with the improvement of people's living standards and diet structure, consumers are paying increased attention to their own health and meat quality. However, for fat-tailed sheep, most of the fat is deposited in the tail, leading to the reduction of fat deposition in other parts of the body, which affects meat quality (7). In modern mutton sheep production systems, tail fat deposition requires higher energy costs. In addition, tail fat accounts for 20% of the carcass weight, which greatly reduces the economic value of the carcass and increases the feeding cost (8). Thus, reducing tail fat deposition has become a research hotspot in sheep genetic improvement.

NIK- and IKKβ-binding protein (NIBP), also known as trafficking protein particle complex 9 (*TRAPPC9*), is a nuclear factor kappa B (NF-κB) signaling pathway regulating factor that has been detected in human nerve cells. It has become clear that the *TRAPP* complex might exist in different forms depending on its specific functions (9). In addition, the NF-κB signaling pathway is the key mediator of cell proliferation, apoptosis, and physiological and pathological events in tumorigenesis (10). Wang et al., conducted genome-wide association studies on Chinese Holstein cows, and the results supported the presence of significant single nucleotide polymorphisms (SNPs), mainly located in *Bos taurus* autosome (BTA) 14 of Chinese Holstein cows, revealing a new candidate gene, *TRAPPC9*, gene related to cow mastitis resistance (11). Another study showed that microcephaly and obesity are common features of *TRAPPC9*-deficient patients (12/23 cases) and summarized this phenotype in a *TRAPPC9*-deficient mouse model (12). Briollais et al. showed that the mean effect of exclusive breastfeeding (EBF) was associated with a 0.06 reduction in the M value of the *TRAPPC9* CpG locus in the first 2 years of life, which resulted in a 0.20 kg/m^2^ reduction in body mass index (BMI) (13). In a study by Liang et al., deleting *TRAPPC9* in mice resulted in the weight of mice increasing significantly, and it was concluded that the loss of *TRAPPC9* function led to the weight gain (14). Liu et al. showed that *TRAPPC9* is related to body shape traits in pigs, in which it participates in the regulation of bone growth and development and nutrient absorption, and is associated with obesity (15). Insulin receptor substrate p53 (IRSp53), also known as brain-specific angiogenesis inhibitor 1-associated protein 2 (*BAIAP2*), is a multi-domain adapter protein originally identified as a tyrosine protein phosphorylated by the insulin receptor and insulin-like growth factor 1 (IGF-1) receptor (16). Lakshman et al. found that *BAIAP2* was significantly associated with weight loss in participants with chronic obstructive pulmonary disease (COPD) (17). Al-Dokhi showed that adults tend to gain weight as they get older, caused by fat deposits (18). In addition, Lee and Shin reported that *TRAPPC9* and *BAIAP2* were related to fat accumulation of pigs using a genome-wide association study (GWAS) (19). However, to the best of our knowledge, there have been no reports on the association of polymorphisms in *TRAPPC9* and *BAIAP2* with fat deposition related-traits in Hu sheep.

Therefore, in the present study, we analyzed the relationship between the single SNPs of *TRAPPC9* and *BAIAP2* and fat deposition-related traits in Hu sheep. In addition, the expression levels of *TRAPPC9* and *BAIAP2* mRNAs in ten different tissues of Hu sheep and tail adipose tissue of small-tail and big-tail Hu sheep were also investigated. This study provided valuable molecular markers for Hu sheep breeding.

## Materials and Methods

### Experimental Sheep and Extraction of DNA

A total of 1,293 Male Hu sheep were purchased from Jinchang Zhongtian Sheep Industry Co., Ltd., Gansu Zhongsheng Huamei Sheep Industry Development Co., Ltd., Gansu Sanyang Jinyuan Animal Husbandry Co., Ltd., Shandong Runlin Sheep Industry Co., Ltd., and Wuwei Pukang Sheep Industry Co., Ltd. Lambs were immunized according to standard procedures before weaning at 56 days of age. All weaned lambs were raised at Minqin Defu Agriculture Co., Ltd. (Gansu, China). The lamb acclimation period was 14 days, the pre-experiment period was 10 days, and the experimental period was 100 days. The feeding conditions were consistent, including housing, feeding, and drinking water. Hu sheep were fed with pellet feed purchased from Gansu Sanyang Jinyuan Animal Husbandry Co., Ltd. All experimental animals were weighed and slaughtered at 180 days. After slaughter, the weight of tail fat, mesenteric fat, and perirenal fat were measured, and collected, and then stored at −80°C for subsequent RNA extraction. DNA was extracted from blood of 1,293 adult sheep (6 months old) using an EASYPURE Blood Genomic DNA Kit (Transgen Biotech, Beijing, China). DNA was stored in elution buffer (10 mM Tris-HCl, 1 mM EDTA; pH 8.0) at −20°C.

### SNP Identification and Genotyping

PCR primers were designed according to the gene sequences to conduct PCR amplification of *TRAPPC9* (NC_040260.1 Chromosome 9 Reference Oar_rambouillet_v1.0 Primary Assembly) and *BAIAP2* (NC_040262.1 Chromosome 11 Reference Oar_rambouillet_v1.0 Primary Assembly) sequences (Table 1). Using mixed DNA (n = 20), the PCR products were sequenced using Sanger sequencing to determine the SNPs of *TRAPPC9* and *BAIAP2*. The PCR reaction (35 μL) included 17.5 μL of 2 × Easy Taq PCR Super Mix (Transgen), 1.12 μL of each primer (forward and reverse), 1.4 μL of dNTPs, and 14 μL ddH_2_O. The thermal cycling procedure for the *TRAPPC9* gene included 5 min at 94°C; followed by 30 s at 94°C, 30 s at 54°C, and 30 s at 72°C for 35 cycles; with a final extension for 5 min at 72°C. The thermal cycling procedure for the *BAIAP2* gene included 5 min at 94°C; 30 s at 94°C, 30 s at 60°C, and 30 s at 72°C for 35 cycles; with a final extension for 5 min at 72°C. According to previous studies, genotyping was performed using competitive allele-specific fluorescence resonance energy transfer (FRET)-based PCR (KASPar) assays (LGC Genomics, Hoddesdon, UK) (20). The primers used for genotyping are shown in Table 2. In this experiment, the *TRAPPC9* and *BAIAP2* genes of 1,162 and 1,046 individuals, respectively, were successfully genotyped, and 970 individuals were successfully genotyped for both genes.

**Table 1:** Primer pairs for the *TRAPPC9* and *BAIAP2* genes used for qRT-PCR and PCR amplification.

**Gene name**	**Primer name**	**Primer sequence (5^′^-3^′^)**	**GenBank accession number**	**Size**	**Tm (°C)**
*TRAPPC9*	*TRAPPC9*-SNP-F	AGCACATACCCCTTTCGTGA	NC_056062	988 bp	54°C
	*TRAPPC9*-SNP-R	TGGCACACATTTAAACTAGGGA			
*BAIAP2*	*BAIAP2*-SNP-F	GCGTCCCGGTTTACACTGCT	NC_040262	604 bp	60°C
	*BAIAP2*-SNP-R	AGGAACACCTGCTGGACACA			
*β-actin*	*β-actin-F*	TCCGTGACATCAAGGAGAAGC	NM_001009784.3	267 bp	60°C
	*β-actin-R*	CCGTGTTGGCGTAGAGGT			
*TRAPPC9*	*TRAPPC9*-expression-F	GCGGCCAACAGACATCGACCA	XM_042254050.1	197 bp	54°C
	*TRAPPC9*-expression-R	AACTCAATCACCCCGGCGTTC			
*BAIAP2*	*BAIAP2*-expression-F	ACCCGCAGAAATACTCGGACA	XM_024450535.2	152 bp	60°C
	*BAIAP2*-expression-R	GGCGCACTGCTTTTCCACCA			

**Table 2:** KASPar genotyping primers.

**Gene**	**Primer name**	**Primer sequence (5^′^-3^′^)**
*TRAPPC9*	Primer_AlleleX	GAAGGTGACCAAGTTCATGCTGTATATATGTTTTATTTACAAAAATGAGAGCG
	Primer_AlleleY	GAAGGTCGGAGTCAACGGATTATGTATATATGTTTTATTTACAAAAATGAGAGCA
	Primer_Common	GTGCACGCAGCAAGCTGCAGAAA
*BAIAP2*	Primer_AlleleX	GAAGGTGACCAAGTTCATGCTCAGAGTGAGCGGAGGCCCTT
	Primer_AlleleY	GAAGGTCGGAGTCAACGGATTGAGTGAGCGGAGGCCCTC
	Primer_Common	ACAGCAACCTGGCACTAGACCG

### Expression Features of *TRAPPC9* and *BAIAP2* Using Quantitative Real-Time Reverse Transcription PCR

The total RNA of each tissue was extracted using Transzol (Transgen) and reverse transcribed into cDNA using an Evo M-MLV RT Kit with gDNA Clean for qPCR (Accurate Biotechnology Co., Ltd, Hunan, China) following the manufacturer's protocols. Six 180-day-old Hu sheep were randomly selected to analyze the expression levels of *TRAPPC9* and *BAIAP2* mRNA in ten tissues (heart, liver, spleen, lung, kidney, rumen, duodenum, muscle, lymph, and tail fat, *n* = 4 for each tissue). In addition, the mRNA expression levels of *TRAPPC9* and *BAIAP2* in the tail adipose tissue of six small-tail and six big-tail Hu sheep were detected. We assessed the tail fat deposition traits of the sheep, which are shown in Table 3. The mRNA sequences of sheep *TRAPPC9* (XM_042254050.1 Chromosome 9 Reference Oar_rambouillet_v1.0 Primary Assembly) and *BAIAP2* (XM_024450535.2 Chromosome 11 Reference Oar_rambouillet_v1.0 Primary Assembly), were used as templates. Specific primer pairs used for detecting *TRAPPC9* and *BAIAP2* expression were designed using Oligo 7.0 software, which the expected band sizes are 197 bp and 152 bp, respectively. β*-actin* (GenBank Accession no. NM_001009784.3) as reference gene (Table 1). The quantitative real-time PCR (qPCR) step of the qRT-PCR protocol was carried out at 94°C for 3 min; followed by 40 cycles of 15 s at 94°C, the optimum annealing temperature for 15 s, and 72°C for 20 s; with a final extension at 72°C for 5 min. The 2^−Δ*ΔCT*^ method was used to analyze the data (21).

**Table 3:** Performance of the sheep used in qRT-PCR.

	**Small-tail group**	**Big-tail group**	***P*** **-value**
Traits	6	6	
The weight of tail fat	0.572 ± 0.138	1.917 ± 0.138	<0.01
The relative weight of tail fat (body weight)	0.018 ± 0.002	0.036 ± 0.002	<0.01
The relative weight of tail fat (carcass weight)	0.034 ± 0.003	0.065 ± 0.003	<0.01

*Vales for the phenotypic data are shown as the mean ± standard error (SE). P-value as calculated using a t-test*.

### Statistical Analysis

The association analysis between genotypes and the fat deposition-related traits was performed using a general linear model program, which was defined as:


$$\begin{array}{l} \;Y_{ijk}=\text{μ}+G_{i}+F_{j}+{\text{ε}}_{ijk}\\ Y_{imjkn}=\text{μ}+G_{i}+G_{m}+F_{j}+C_{n}+\;{\text{ε}}_{imjkn} \end{array}$$

Where, Y_*ijk*_ and Y_*imjkn*_ are the phenotypic observation value of the tail fat deposition traits, μ is the mean, G_*i*_ and G_*m*_ is the effect of the ith and mth genotypes, F_*j*_ represents the farm effect (*j* = 1, 2……5), C_*n*_ refers to the effect of combination, and ε_*ijk*_ and ε_*imjkn*_ are the residuals corresponding to the observed trait values. A *P*-value < 0.05 or a *P*-value < 0.01 were regarded as statistically significant and highly significant, respectively. The genotypic frequency and allele frequency were calculated. SPSS v.23 software was used for all statistical analyses (IBM Corp., Armonk, NY, USA).

## Results

### Descriptive Statistics of Fat Deposition Related Traits

In the present study, the fat deposition-related traits of all lambs (*n* = 1,293) were measured after slaughter at 180 days of age, and the descriptive statistics for the phenotypes of all the traits are shown in Table 4. The coefficient of variation for all traits was great than 24%, among which the variation coefficient of tail fat weight, perirenal fat weight, and mesenteric fat weight were 30.28, 47.43, and 38.34%, respectively. These results suggested the fat deposition traits have marked phenotypic variation in the experimental population.

**Table 4:** Descriptive statistics of fat traits.

**Items**	**Min**	**Max**	**Mean**	**SD**	**CV (%)**
The weight of tail fat	0.31	3.23	1.50	0.01	30%
The relative weight of tail fat (carcass weight)	0.01	0.07	0.03	0.00	26%
The relative weight of tail fat (body weight)	0.02	0.15	0.06	0.00	25%
The weight of perirenal fat	0.07	2.12	0.63	0.01	47%
The relative weight of perirenal fat (carcass weight)	0.00	0.07	0.02	0.00	42%
The relative weight of perirenal fat (body weight)	0.00	0.04	0.01	0.00	42%
The weight of mesenteric fat	0.15	3.14	1.08	0.01	38%
The relative weight of mesenteric fat (carcass weight)	0.01	0.11	0.04	0.00	33%
The relative weight of mesenteric fat (body weight)	0.00	0.06	0.02	0.00	33%

### SNP Scanning of Sheep *TRAPPC9* and *BAIAP2* Genes

**Figure 1:**
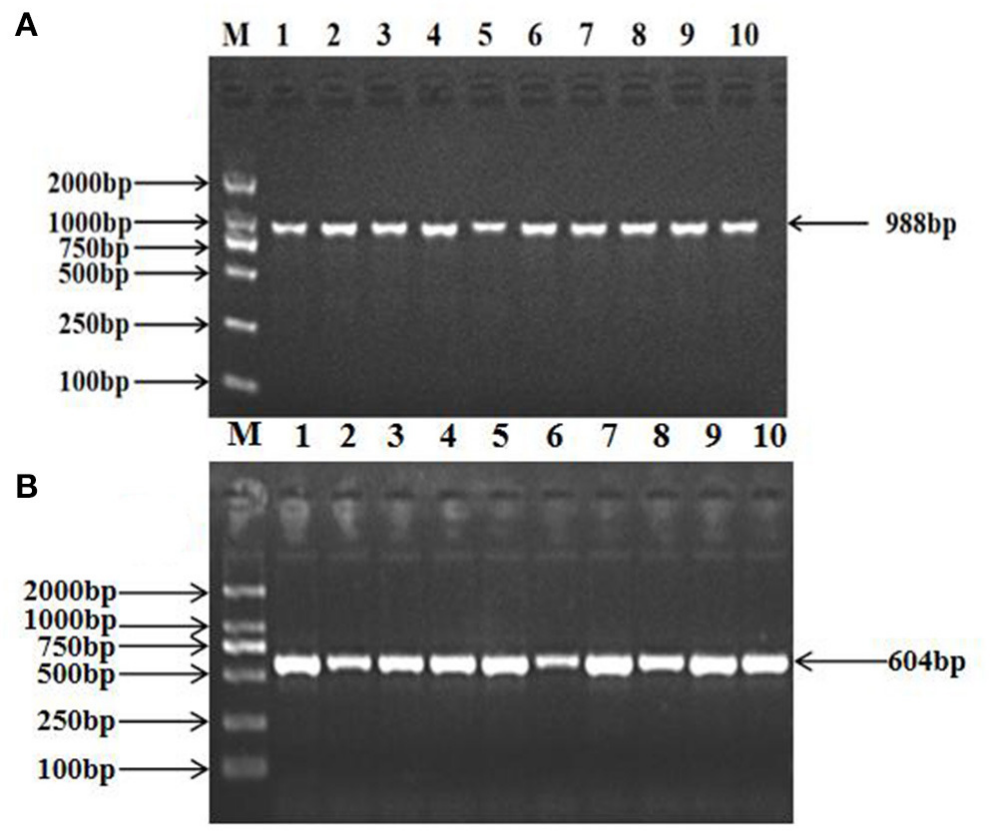
PCR amplification of the target fragments of the ovine *TRAPPC9* **(A)** and *BAIAP2* **(B)** genes. M: DL2000 DNA Marker; 1–10: PCR products.

**Figure 2:**
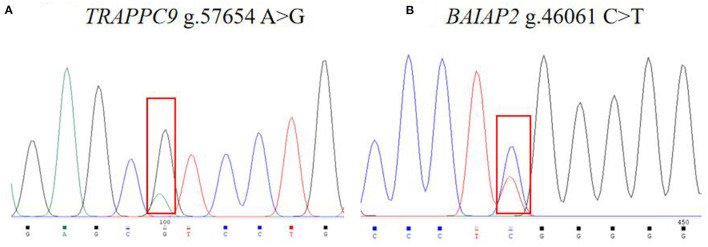
Image of the sequencing peaks of sheep *TRAPPC9* **(A)** and *BAIAP2* **(B)** loci.

The 988 bp fragment of *TRAPPC9* and the 604 bp fragment of *BAIAP2* were amplified using the primers shown in Table 2 (Figure 1). The amplified PCR products were sequenced by Tsingke Ltd. (Xi'an, China). A new mutation was found in *TRAPPC9*, located in intron 10 (g.57654 A > G), and a new mutation was also found in *BAIAP2*, located in intron 6 (g.46061 C > T, Figure 2).

### Genotyping, and Genotype and Allele Frequency Analysis

**Figure 3:**
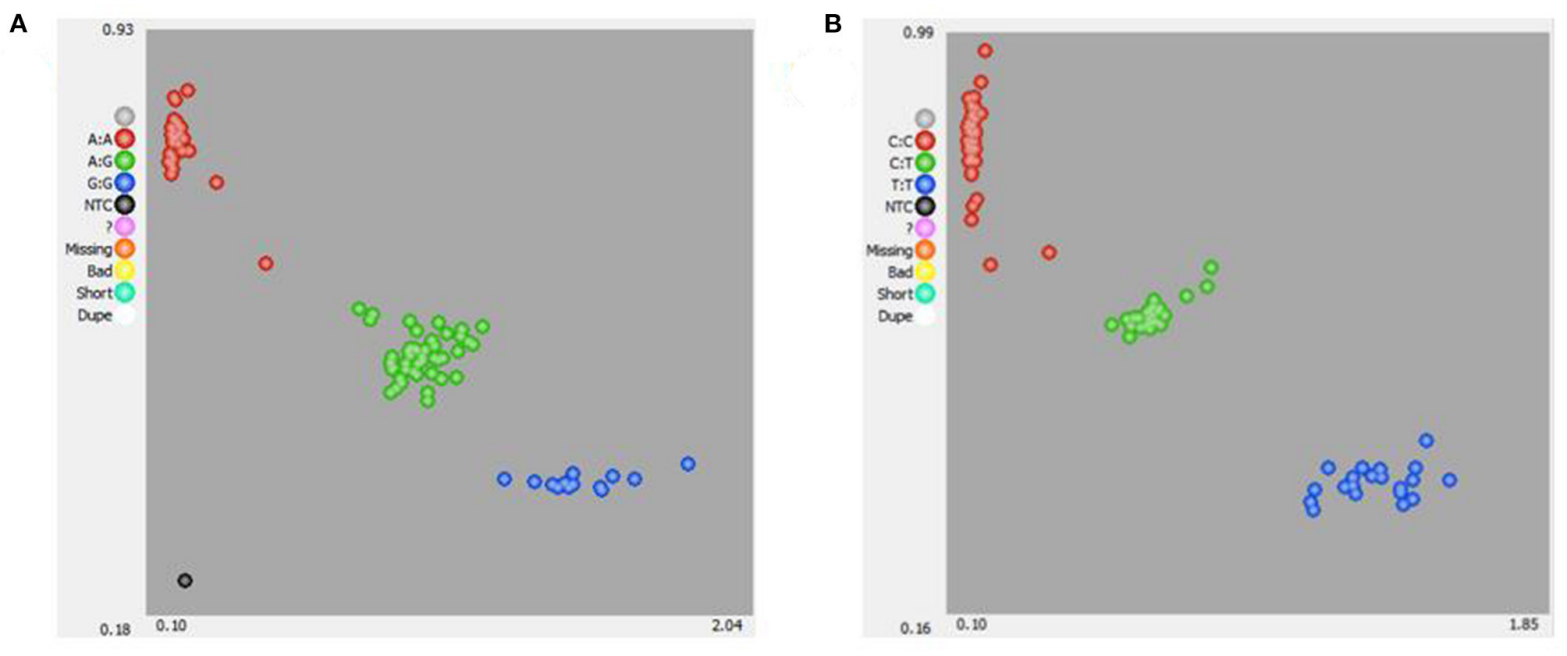
KASPar-based single nucleotide polymorphism (SNP) genotyping of sheep *TRAPPC9* g.57654 A > G **(A)** and *BAIAP2* g.460 C > T **(B)**.

KASPar analysis was used to genotype the two SNPs, and three genotypes of the two genes were determined: AA, AG, and GG (*TRAPPC9*) and CC, CT, and TT (*BAIAP2*) (Figure 3). The genetic parameters of the SNPs recognized in Hu sheep at *TRAPPC9* g.57654 A > G and *BAIAP2* g.46061 C > T loci were calculated (Table 5). For the *TRAPPC9* g.57654 A > G locus, the genotype frequencies were 0.45, 0.44, and 0.11, respectively. The results of allele frequency analysis showed that the frequency of the A allele was 0.67, which accounted for the highest proportion in the population. For the *BAIAP2* g.46061 C > T locus, the genotype frequencies were 0.58, 0.29, and 0.13, respectively. The results of allele frequency analysis showed that the frequency of the C allele was 0.73, which accounted for the highest proportion in the population. The polymorphism information content (PIC), effective allele number (Ne), expected homozygosity (Ho), expected heterozygosity (He), and the *P*-value of the Hardy-Weinberg equilibrium (PHWE) of *TRAPPC9* were 0.34, 1.79, 0.56, 0.44, and 0.84, respectively, and the PIC, Ne, Ho, He, and PHWE of *BAIAP2* were 0.31, 1.64, 0.61, 0.39, and 0, respectively (Table 5).

**Table 5:** The genotype frequency, allele frequency, and genetic diversity of the *TRAPPC9* and *BAIAP2* SNP sites.

**Loci**	**Genotype**	**Genotype frequency**	**Allele**	**Allele frequency**	**Ne**	**Ho**	**He**	**PIC**	**PHWE**
*TRAPPC9* g.57654A > G	AA (527)	0.45	A	0.67	1.79	0.56	0.44	0.34	0.84
	AG (509)	0.44							
	GG (126)	0.11	G	0.33					
*BAIAP2* g.46061A > G	CC (610)	0.58	C	0.73	1.64	0.61	0.39	0.31	0.00
	CT (299)	0.29							
	TT (137)	0.13	T	0.27					

*Expected heterozygosity (He), expected homozygosity (Ho), effective allele number (Ne), polymorphism information content (PIC), and the P-value of the Hardy-Weinberg equilibrium (PHWE)*.

### Association Analysis Between *TRAPPC9* and *BAIAP2* Genes and Fat Deposition Traits in Hu Sheep

The association analysis showed that the *TRAPPC9* g.57654 A > G gene polymorphism correlated significantly with tail fat deposition traits (*P* < 0.05). The tail fat weight, relative tail fat weight (body weight), and relative tail fat weight (carcass) of the GG genotype were significantly lower than those of the AA genotype (*P* < 0.05). Therefore, GG was a significant genotype associated with tail fat deposition in Hu sheep. The *BAIAP2* g.46061 C > T gene polymorphism also correlated significantly with tail fat deposition traits (*P* < 0.05). The tail fat weight, relative tail fat weight (body weight), and relative tail fat weight (carcass) of the CT genotype were significantly lower than those of the TT genotype (*P* < 0.05). However, no significant association was observed between the genes and perirenal fat weight and mesenteric fat weight (*P* > 0.05) (Table 6).

**Table 6:** Analysis of the associations of sheep *TRAPPC*9 g.57654 A > G and *BAIAP2* g.46061 C > T SNPs.

	***TRAPPC9*** **g.57654 A > G**			***BAIAP2*** **g.46061 C > T**		
**Items**	**AA**	**AG**	**GG**	**CC**	**CT**	**TT**
No.	527	509	126	610	299	137
The weight of tail fat	1.546 ± 0.02^a^	1.484 ± 0.02^ab^	1.413 ± 0.04^b^	1.503 ± 0.018^ab^	1.45 ± 0.026^b^	1.57 ± 0.038^a^
The relative weight of tail fat (carcass weight)	0.059 ± 0.001^a^	0.058 ± 0.001^ab^	0.056 ± 0.001^b^	0.032 ± 0.00^ab^	0.031 ± 0.00^b^	0.033 ± 0.001^a^
The relative weight of tail fat (body weight)	0.032 ± 0.00^a^	0.031 ± 0.00^ab^	0.03 ± 0.001^b^	0.058 ± 0.001^ab^	0.057 ± 0.001^b^	0.061 ± 0.001^a^
The weight of perirenal fat	0.638 ± 0.013	0.623 ± 0.013	0.619 ± 0.027	0.627 ± 0.012	0.609 ± 0.017	0.646 ± 0.025
The relative weight of perirenal fat (carcass weight)	0.024 ± 0.00	0.024 ± 0.00	0.024 ± 0.001	0.024 ± 0.00	0.023 ± 0.001	0.025 ± 0.001
The relative weight of perirenal fat (body weight)	0.013 ± 0.00	0.013 ± 0.00	0.013 ± 0.00	0.013 ± 0.00	0.013 ± 0.00	0.014 ± 0.00
The weight of mesenteric fat	1.106 ± 0.018	1.066 ± 0.018	1.052 ± 0.037	1.09 ± 0.017	1.057 ± 0.024	1.093 ± 0.036
The relative weight of mesenteric fat (carcass weight)	0.042 ± 0.001	0.042 ± 0.001	0.041 ± 0.001	0.042 ± 0.001	0.041 ± 0.001	0.042 ± 0.001
The relative weight of mesenteric fat (body weight)	0.023 ± 0.00	0.022 ± 0.00	0.022 ± 0.001	0.023 ± 0.00	0.022 ± 0.00	0.023 ± 0.001

*SNP, single nucleotide polymorphism*.

*Values for the phenotypic data are shown as the mean ± standard error. Different superscript lowercase letters against values in the same column indicate significant differences (P < 0.05) or extremely significant differences (P < 0.01)*.

### Association Analysis of the Combination Genotypes of the *TRAPPC9* and *BAIAP2* Genes With the Tail Fat Deposition Traits of the Hu Sheep

The comprehensive effects of *TRAPPC9* g.57654 A > G and *BAIAP2* g.46061 C > T on tail fat deposition traits were analyzed (Table 7). The results showed that tail fat weight, tail fat relative weight (body weight), and tail fat relative weight (carcass) of the GG^*TRAPPC*9^/CT^*BAIAP*2^, AG^*TRAPPC*9^/CC^*BAIAP*2^, GG^*TRAPPC*9^/CC^*BAIAP*2^, and AA^*TRAPPC*9^/CC^*BAIAP*2^ genotype were significantly lower than those of the AA^*TRAPPC*9^/TT^*BAIAP*2^, and AA^*TRAPPC*9^/CT^*BAIAP*2^ combined genotypes (*P* < 0.05). The perirenal fat weight, perirenal fat relative weight (body weight), and perirenal fat relative weight (carcass) of the GG^*TRAPPC*9^/CT^*BAIAP*2^ genotype were significantly lower than those of the AA^*TRAPPC*9^/TT^*BAIAP*2^, AA^*TRAPPC*9^/CT^*BAIAP*2^, AG^*TRAPPC*9^/TT^*BAIAP*2^, and GG^*TRAPPC*9^/CC^*BAIAP*2^ combined genotypes (*P* < 0.05). The mesenteric fat weight of the AG^*TRAPPC*9^/CT^*BAIAP*2^ genotype was significantly lower than that of the AA^*TRAPPC*9^/CT^*BAIAP*2^ genotype (*P* < 0.05), but not significantly different compared with other genotypes, and there was no significant difference between the mesenteric fat relative weight (body weight) and the mesenteric fat relative weight (carcass) among the genotypes (*P* > 0.05).

**Table 7:** Analysis of the associations of combined genotypes at the *TRAPPC9* and *BAIAP2* loci and sheep tail fat deposition traits.

**Items**	**AA^*TRAPPC*9^/**	**AA^*TRAPPC*9^/**	**AG^*TRAPPC*9^/**	**AG^*TRAPPC*9^/**	**AA^*TRAPPC*9^/**	**GG^*TRAPPC*9^/**	**GG^*TRAPPC*9^/**	**AG^*TRAPPC*9^/**	**GG^*TRAPPC*9^/**
	**TT^*BAIAP*2^**	**CT^*BAIAP*2^**	**TT^*BAIAP*2^**	**CT^*BAIAP*2^**	**CC^*BAIAP*2^**	**CC^*BAIAP*2^**	**TT^*BAIAP*2^**	**CC^*BAIAP*2^**	**CT^*BAIAP*2^**
No.	68	129	52	111	250	56	6	249	49
The weight of tail fat	1.602 ± 0.054^a^	1.561 ± 0.028^a^	1.522 ± 0.062^ab^	1.477 ± 0.042^abc^	1.458 ± 0.039^bc^	1.454 ± 0.059^bc^	1.451 ± 0.182^bc^	1.439 ± 0.028^bc^	1.332 ± 0.064^c^
The relative weight of tail fat (body weight)	0.033 ± 0.001^a^	0.033 ± 0.001^a^	0.032 ± 0.001^ab^	0.032 ± 0.001^ab^	0.03 ± 0.001^b^	0.03 ± 0.001^b^	0.033 ± 0.003^ab^	0.031 ± 0.001^b^	0.028 ± 0.001^b^
The relative weight of tail fat (carcass weight)	0.061 ± 0.002^a^	0.06 ± 0.001^a^	0.06 ± 0.002^ab^	0.058 ± 0.001^ab^	0.057 ± 0.001^bc^	0.056 ± 0.002^bc^	0.061 ± 0.006^a^	0.057 ± 0.001^bc^	0.053 ± 0.002^c^
The weight of perirenal fat	0.656 ± 0.035^a^	0.639 ± 0.019^a^	0.658 ± 0.041^a^	0.601 ± 0.028^ab^	0.623 ± 0.026^ab^	0.664 ± 0.039^a^	0.655 ± 0.119^a^	0.601 ± 0.019^ab^	0.542 ± 0.042^b^
The relative weight of perirenal fat (body weight)	0.014 ± 0.001^a^	0.013 ± 0^a^	0.014 ± 0.001^a^	0.013 ± 0.001^ab^	0.013 ± 0^ab^	0.014 ± 0.001^a^	0.015 ± 0.002^ab^	0.013 ± 0^ab^	0.011 ± 0.001^b^
The relative weight of perirenal fat (carcass weight)	0.025 ± 0.001^a^	0.024 ± 0.001^a^	0.026 ± 0.001^a^	0.023 ± 0.001^ab^	0.023 ± 0.001^ab^	0.025 ± 0.001^a^	0.027 ± 0.004^ab^	0.024 ± 0.001^ab^	0.021 ± 0.001^b^
The weight of mesenteric fat	1.133 ± 0.05^ab^	1.117 ± 0.026^a^	1.035 ± 0.057^ab^	1.018 ± 0.039^b^	1.063 ± 0.036^ab^	1.118 ± 0.055^ab^	0.957 ± 0.169^ab^	1.053 ± 0.026^ab^	1 ± 0.059^ab^
The relative weight of mesenteric fat (body weight)	0.024 ± 0.001	0.023 ± 0.00	0.022 ± 0.001	0.022 ± 0.001	0.022 ± 0.001	0.023 ± 0.001	0.022 ± 0.003	0.022 ± 0.00	0.021 ± 0.001
The relative weight of mesenteric fat (carcass weight)	0.043 ± 0.002	0.043 ± 0.001	0.041 ± 0.002	0.04 ± 0.001	0.041 ± 0.001	0.043 ± 0.002	0.04 ± 0.006	0.042 ± 0.001	0.039 ± 0.002

*Different superscript lowercase letters against values in the same column indicate significant differences (P < 0.05) or extremely significant differences (P < 0.01) (Tukey test)*.

### Expression Profile Analysis

**Figure 4:**
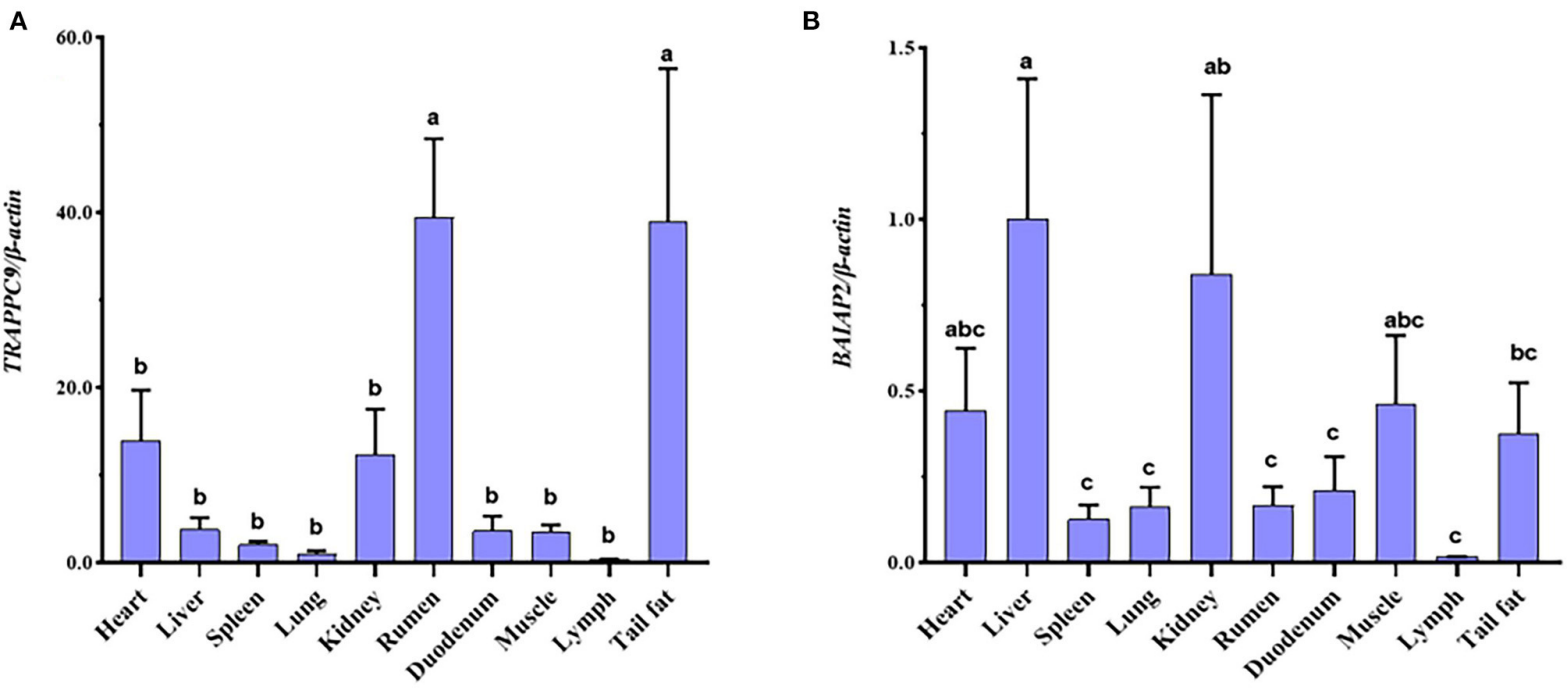
*TRAPPC9* mRNA expression profile in sheep tissues **(A)**. *BAIAP2* mRNA expression profile in sheep tissues **(B)**. Different lowercase letters indicate a significant difference (*P* < 0.05).

The expression levels of *TRAPPC9* and *BAIAP2* in the tail fat, lymph, muscle, duodenum, rumen, kidney, lung, spleen, liver, and heart were detected using qRT-PCR. The results showed that *TRAPPC9* and *BAIAP2* were expressed in these ten tissues. Moreover, the expression levels of *TRAPPC9* in the tail fat and rumen were significantly higher than those in the other tissues (*P* < 0.05), and the expression levels of *BAIAP2* in the heart, liver, kidney, muscle, and tail fat were significantly higher than those in the other tissues (*P* < 0.05; Figure 4).

### Expression of *TRAPPC9* and *BAIAP2* Genes in Extreme Tail Fat Tissue Types

**Figure 5:**
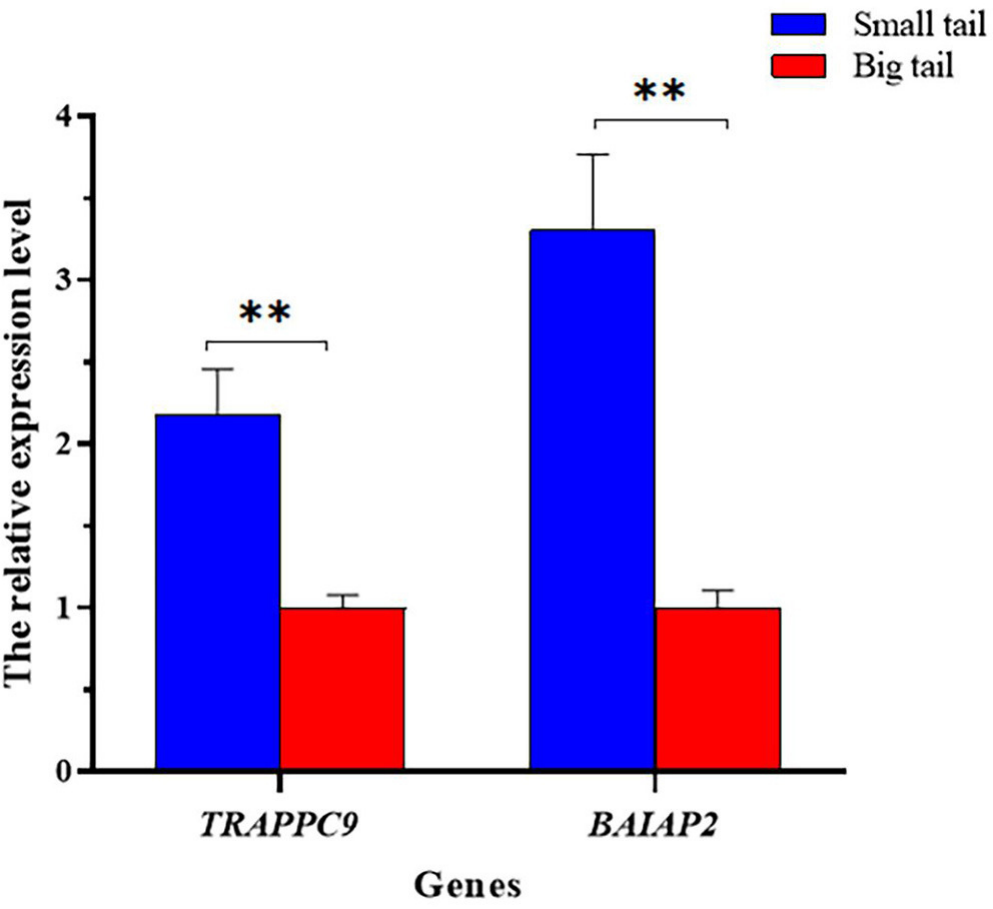
The relative *TRAPPC9* and *BAIAP2* mRNA expression levels between the small-tail and big-tail groups. Asterisks indicate a significant differences between the small- and big-tail groups (*P* < 0.05), double asterisks indicate a very significant differences between the small-tail and big-tail groups (*P* < 0.01).

The mRNA expression levels of *TRAPPC9* and *BAIAP2* between small-tailed sheep and big-tailed sheep were analyzed quantitatively using qRT-PCR. The results showed that the expression levels of *TRAPPC9* and *BAIAP2* in the small-tailed group were significantly higher than those in the big-tailed group (*P* < 0.01; Figure 5).

## Discussion

Fat tails in sheep are perceived to have developed following domestication and are a valuable energy reserve for the animals during migration and winter (4, 22). However, fat tails might affect reproduction and fattening, thereby increasing the cost of raising sheep and reducing their economic value (23, 24). Fat deposition in sheep tails is the result of a complex mechanism. Previous studies have conducted several investigations into the inheritance of fat tails (25); nevertheless, the mechanisms of the genes affecting fat deposition in fat tail sheep remain unknown. *TRAPPC9* is a subunit of the highly conserved protein complex called the transport protein particle (*TRAPP*), a guanine nucleotide exchange factor for rab proteins that operates in secretory, endocytic, and autophagic pathways (26). Over half of the patients with *TRAPPC9* mutations are reported to present different degrees of obesity (27). Hnoonual et al. (12) found that the deletion of the *TRAPPC9* gene leads to obesity. *BAIAP2*, which is located on 17q25 and encodes brain-specific angiogenesis inhibitor 1-associated protein 2 (*BAIAP2*), has been suggested to be involved in cerebral asymmetry (28). In a study by Lakshman et al. (17), the *BAIAP2* gene was significantly associated with weight change in patients with chronic obstructive pulmonary disease (COPD). *TRAPPC9* and *BAIAP2* were implicated in fat accumulation by a GWAS (19). In this study, Two intronic mutations were detected in the *TRAPPC9* and *BAIAP2* genes, respectively. Recent studies suggest intronic mutations can change protein levels or protein conformation by altering regulatory splice sites, mRNA stability, miRNA binding sites, or translation efficiency (29–33). To further investigate the effect of these two intronic mutations on sheep phenotype, we analyzed the potential association between genotype and fat deposition. The results showed that *TRAPPC9* g.57654 A > G and *BAIAP2* g.46061 C > T were both associated significantly with tail fat deposition.

We detected that *TRAPPC9* and *BAIAP2* were expressed in all ten tissues of Hu sheep tested. Zhang et al. showed that *TRAPPC9* was highly expressed in muscles and kidneys of human tissues; showed low expression in the heart, brain, and placenta; and was weakly expressed in the thymus and spleen (34). In Abbott et al.'s study, the tissue distribution of *BAIAP2* mRNA was determined by northern blotting, appearing mainly in the heart, brain, spleen, lung, liver, kidney, and testis (35). Our results are consistent with those of previous studies. *TRAPPC9* was significantly expressed in the rumen and tail fat (*P* < 0.05). However, studies on the *TRAPPC9* gene in the rumen are rarely reported. This gene may be related to the growth and development of the rumen, but the specific mechanism still needs further study. Usman et al. found through research that mouse *TRAPPC9* gene deficiency affects the proliferation and differentiation ability of adipose stem cells (ASCs) (36). Our qRT-PCR results also confirmed this view. Therefore, we speculate that *TRAPPC9* has the same function in sheep.

The expression of *BAIAP2* in the liver was significantly higher than that in other tissues (*P* < 0.05). Previous studies have found that the *BAIAP2* is significantly expressed in the liver (37–39). The liver plays a pivotal role in regulating the metabolism of fatty acids (FA) and their neutral storage form, triglycerides (TGs) (40, 41). A study identified that interactions between adipose tissue and the liver might play a role in the development of non-alcoholic fatty liver disease (42). Therefore, we speculated that *BAIAP2* might affect tail fat deposition by regulating FA synthesis.

To verify the potential roles of *TRAPPC9* and *BAIAP2* in ovine tail fat deposition, we analyzed the mRNA expression levels of *TRAPPC9* and *BAIAP2* between small-tail and big-tail sheep. The results showed that the expression levels of *TRAPPC9* and *BAIAP2* in the small-tailed group were significantly higher than those in the large-tailed group (*P* < 0.01). Therefore, we speculated the polymorphic loci in *TRAPPC9* and *BAIAP2* might represent important genetic markers in studies designed to reduce tail fat deposition in Hu sheep, and also provide clues for further search for causal mutations of tail fat deposition. However, the regulatory mechanism of *TRAPPC9* and *BAIAP2* on tail fat deposition in Hu sheep require further study.

## Conclusions

In the present study, two novel SNPs in the *TRAPPC9* and *BAIAP2* genes were identified. Association analysis indicated that the two SNPs were significantly related to tail fat weight-related traits of Hu sheep. In addition, the two genes were expressed widely in ten tissues of Hu sheep, and the expression levels of *TRAPPC9* and *BAIAP2* in the small-tail group were significantly higher than those in the big-tail group. Thus, we speculated that the polymorphic loci in *TRAPPC9* and *BAIAP2* might be used as genetic markers of tail fat deposition in Hu sheep.

## Data Availability Statement

The datasets presented in this study can be found in online repositories. The names of the repository/repositories and accession number(s) can be found in the article/Supplementary Material.

## Ethics Statement

All experiments in this study were authorized and approved by the Animal Welfare and Ethics Committee of Gansu Agricultural University, and carried out in accordance with the regulations of the Standing Committee of the People's Congress of Gansu Province. License No. 2012-2-159.

## Author Contributions

WW, XZh, and PC were involved in the design and design of the experiment. CLi, LZ, DX, JC, YZhan, XL, XZe, YZhao, WL, JW, RZ, CLin, JL, and BZ collected the experimental samples. DZ, YH, ZM, XW, XY, and PC conducted the data analysis. PC wrote the paper. XZh revised the manuscript. All authors contributed to the article and approved the submitted version.

## Funding

This work was supported by the National Key Research and Development Program of China (No. 2021YFD1300901), the National Natural Science Foundation of China (31960653), and the Key Research and Development Project of Gansu Province, China (20YF3NA012).

## Conflict of Interest

The authors declare that the research was conducted in the absence of any commercial or financial relationships that could be construed as a potential conflict of interest.

## Publisher's Note

All claims expressed in this article are solely those of the authors and do not necessarily represent those of their affiliated organizations, or those of the publisher, the editors and the reviewers. Any product that may be evaluated in this article, or claim that may be made by its manufacturer, is not guaranteed or endorsed by the publisher.
